# Geometrical Frustration in Interleukin-33 Decouples the Dynamics of the Functional Element from the Folding Transition State Ensemble

**DOI:** 10.1371/journal.pone.0144067

**Published:** 2015-12-02

**Authors:** Kaitlin M. Fisher, Ellinor Haglund, Jeffrey K. Noel, Kendra L. Hailey, José N. Onuchic, Patricia A. Jennings

**Affiliations:** 1 Department of Chemistry and Biochemistry, University of California at San Diego (UCSD), La Jolla, CA, United States of America; 2 Center for Theoretical Biological Physics (CTBP) and Department of Physics, University of California at San Diego (UCSD), La Jolla, CA, United States of America; 3 Center for Theoretical Biological Physics (CTBP) and Department of Physics and Astronomy, Chemistry and Biochemistry and Cell Biology, Rice University, Houston, TX, United States of America; Universidad de Granada, SPAIN

## Abstract

Interleukin-33 (IL-33) is currently the focus of multiple investigations into targeting pernicious inflammatory disorders. This mediator of inflammation plays a prevalent role in chronic disorders such as asthma, rheumatoid arthritis, and progressive heart disease. In order to better understand the possible link between the folding free energy landscape and functional regions in IL-33, a combined experimental and theoretical approach was applied. IL-33 is a pseudo- symmetrical protein composed of three distinct structural elements that complicate the folding mechanism due to competition for nucleation on the dominant folding route. Trefoil 1 constitutes the majority of the binding interface with the receptor whereas Trefoils 2 and 3 provide the stable scaffold to anchor Trefoil 1. We identified that IL-33 folds with a three-state mechanism, leading to a rollover in the refolding arm of its chevron plots in strongly native conditions. In addition, there is a second slower refolding phase that exhibits the same rollover suggesting similar limitations in folding along parallel routes. Characterization of the intermediate state and the rate limiting steps required for folding suggests that the rollover is attributable to a moving transition state, shifting from a post- to pre-intermediate transition state as you move from strongly native conditions to the midpoint of the transition. On a structural level, we found that initially, all independent Trefoil units fold equally well until a Q_CA_ of 0.35 when Trefoil 1 will backtrack in order to allow Trefoils 2 and 3 to fold in the intermediate state, creating a stable scaffold for Trefoil 1 to fold onto during the final folding transition. The formation of this intermediate state and subsequent moving transition state is a result of balancing the difficulty in folding the functionally important Trefoil 1 onto the remainder of the protein. Taken together our results indicate that the functional element of the protein is geometrically frustrated, requiring the more stable elements to fold first, acting as a scaffold for docking of the functional element to allow productive folding to the native state.

## Introduction

Chronic inflammatory disorders, to date, are very difficult to treat effectively and lead to a variety of damaging pathologies. Diseases associated with inflammation affect millions and yet the treatments currently available are primarily palliative measures [1]. Interleukin-33 (IL-33) is a pro-inflammatory cytokine predominately expressed in epithelial cells as an alarmin and is responsible for activating a variety of cells including mast cells, CD8^+^ T cells, and hematopoietic cells [2,3]. IL-33 acts primarily as a mediator of inflammation and at this time is being actively pursued as a drug target for diseases including asthma, heart disease, cancer [4–6], and rheumatoid arthritis [7,8]. The pursuit of IL-33 as a drug target has potential to treat the inflammatory pathways responsible for causing long lasting damage from chronic inflammation more directly [9].

**Fig 1:**
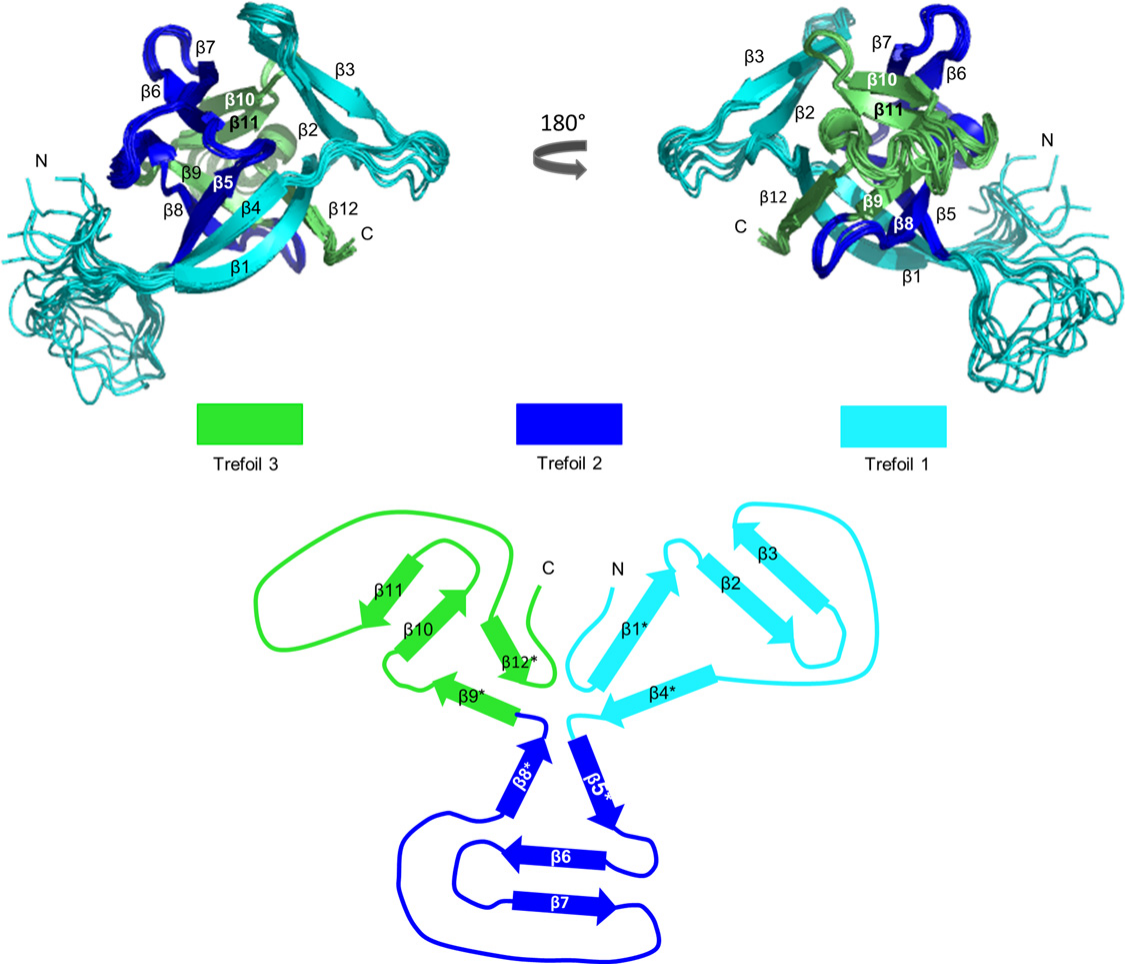
The Topology of Interleukin-33. (Top) The 20 overlaid NMR structures of IL-33 (PDB code 2KLL) are displayed in two orientations as indicated. Trefoil 1 is highlighted in cyan, Trefoil 2 in blue, and Trefoil 3 in green. (Bottom) A representative two-dimensional splay diagram of IL-33 from a top view of the protein. Barrel strands are indicated by asterisks and β-hairpin cap strands are on the periphery of the diagram. Trefoil 1 is composed of strands 1–4, Trefoil 2 strands 5–8, and Trefoil 3 strands 9–12.

IL-33 has a β-trefoil fold that represents a shared motif with other members of the Interleukin-1 family (IL) such as IL-1β and IL-1ra [10,11] and with other β-trefoil proteins such as Human Fibroblast Growth Factor-1 (hFGF-1) and Hisactophilin [12]. The β-trefoil motif is characterized by a three fold pseudo-symmetrical structure. It is composed of three units of four β-strands that fold to form three β-β-β-loop-β elements (Fig 1). The pseudo-symmetry in the final fold may help in the propagation of allostery across the structure as seen in other beta-barrel like proteins [13,14]. Interestingly, the first pseudo-symmetrical element (Trefoil 1) constitutes the large binding interface responsible for engaging IL-33 to its conjugate cell surface receptor ST2, representing the first critical step in formation of the active heterotrimeric signaling complex [15]. The binding of IL-33 to ST2 allows for binding of a secondary accessory receptor to form a ternary signaling complex [16]. IL-33, as a newer member within this family, has not yet been fully biophysically characterized. Understanding the folding free energy landscape is important in terms of the functionality of IL-33, specifically the influence of each trefoil on the biological activity as it pertains to drug design and targeting [17].

The folding route of a protein can illuminate the underlying causes of disease states by investigating the specific events that lead to a functional native protein. The simplest folding landscape is through a two-state mechanism, in which the protein folds directly from the denatured state (D) to the native state (N) without populating any states in between [18]. Two-state folding behavior gives rise to a characteristic V-shaped, linear chevron plot [18]. Proteins can also fold through more complicated mechanisms wherein intermediate states (I) may be populated before reaching the fully native state [19–21]. The complicated folding kinetics of IL-33 involves a rollover and additional second slower phase of folding in strongly native conditions. The folding route of IL-33 required systematic evaluation to identify and characterize the exact source of the more complicated folding kinetics. We used a combined experimental and theoretical approach to investigate the folding free energy landscape in order to fully characterize the novel folding route of this mediator of inflammation.

Our combined results suggest that the rollover in IL-33 is a consequence of an intermediate state along the folding route. The rate-limiting step for folding moves from a pre-intermediate to post-intermediate transition state, causing differing denaturant dependencies and varying slopes for the refolding rates. The pseudo-symmetrical structure of IL-33 not only requires the formation of an intermediate state, it also leads to backtracking in Trefoil 1 due to competition between symmetrical folding nucleation sites. Geometrical frustration, mediated by the geometric accessibility of native contacts, in Trefoil 1 creates bottlenecks on the free energy landscape, constraining it to fold last. As the functional element, Trefoil 1 folding last imparts a unique flexibility to its structure, allowing it to be malleable without affecting the integrity of Trefoils 2 and 3. Interestingly, we see an additional second slower phase that also exhibits a rollover. The observation of this phase is consistent with the hypothesis of parallel refolding routes that are dependent upon the adjustment of contacts within Trefoils 2 and 3 as Trefoil 1 is constrained to a specific geometric orientation for folding to the native basin. Our work shows that geometrical frustration and preservation of the functional element in IL-33 is a strong mediator for the complex experimental kinetics associated with the dominant folding route of this protein.

## Results

### The Structure of Interleukin-33

IL-33, as a member of the IL-1 family of proteins, has the characteristics of a β-trefoil fold. This fold is composed of twelve β-strands organized into three groups of four β-β-β-loop-β units (Trefoil1, Trefoil 2, and Trefoil 3) characterizing the overall fold. Trefoil 1 is composed of β-strands 1 through 4, Trefoil 2 of strands 5 through 8, and Trefoil 3 of strands 9 through 12 (PDB code 2KLL) in Fig 1 [22]. The β-strands are organized in a similar manner within each trefoil. Further highlighting the symmetry of this protein, the contact maps for IL-33 show that the distribution of contacts within each Trefoil is essentially equivalent (208 contacts in Trefoil 1, 221 contacts in Trefoil 2, and 211 contacts in Trefoil 3). However, Trefoil 1 is slightly different in its topology than Trefoils 2 and 3 in that the β-strands are longer, making the sequence of Trefoil 1 overall longer than that of Trefoil 2 and 3. Additionally, Trefoil 1 contains a charged lysine-rich loop between β-strands 3 and 4 and has a large flexible loop bridging β-strands 4 and 5, which constitutes the interface between Trefoil 1 and 2. These unique features of Trefoil 1 make it less symmetrical in nature than the other Trefoils. The more complicated topology and less symmetrical nature of Trefoil 1 versus Trefoil 2 and 3 pairs with the functionality of IL-33: Trefoil 1 acts as the functional element within IL-33, as it has the largest binding interface with its conjugate receptor, ST2 [23].

### Experimental Characterization Shows Evidence for the Formation of an Intermediate State

**Fig 2:**
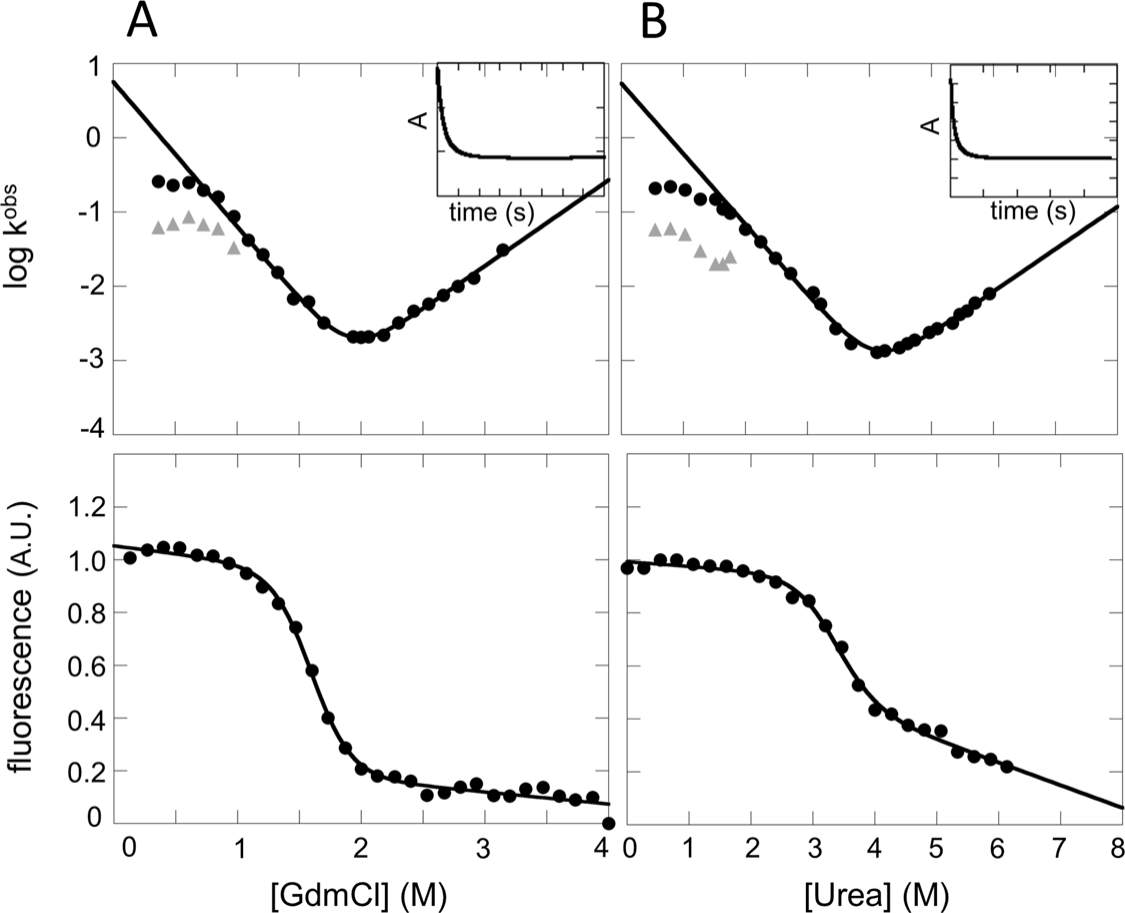
Experimental kinetic and equilibrium folding data for Interleukin-33. (A) The chevron plot of the observed rate of folding and unfolding as a function of final denaturant concentration for IL-33 (upper panel) and the equilibrium titrations as a function of final GdmCl concentration (lower panel). (B) The chevron plot for IL-33 (upper panel) and the equilibrium unfolding curve for IL-33 as a function of final urea concentration (lower panel) constructed as in (A). Representative exponential fits of folding for GdmCl and Urea are shown as inset panels within the chevron plots. Grey triangles within the chevron plots represent the second fitted phase in strongly native conditions. The data are well fit by a single exponential over most denaturant concentrations except in strongly native conditions (<1 M GdmCl and <2 M urea, respectively) where a rollover appears and the refolding rates are fit to a double exponential equation. The total chevron plots are fit to a two-state equation over the linear regime. The equilibrium curves are fitted with a two-state equation.

In order to characterize the folding free energy landscape of IL-33, we performed thermodynamic and kinetic experiments using intrinsic tryptophan fluorescence, utilizing the single Tryptophan 84 located within Trefoil 2 in IL-33, in the presence of both Guanidine Hydrochloride (GdmCl) and Urea. Equilibrium titrations were evaluated (Fig 2A and 2B, bottom panel) to investigate the thermodynamic behavior of IL-33. The equilibrium data fits well to a two-state equation and there is no significant evidence for a thermodynamically stable intermediate state (Table 1). The relative solvent accessible surface area (SASA) can be measured experimentally and calculated as an *m*_D-N_ value (calculated as the difference between the slope of the unfolding arm of the chevron plot and the slope of the folding arm). The *m*_D-N_ values for IL-33 are within experimental error of each other for GdmCl and for Urea calculated from the equilibrium curve and chevron plots, respectively. The *m*_D-N_ values are in the expected range for a protein the size of IL-33 [24].

**Table 1:** Equilibrium Titrations.

	*m*_D-N_ (kcal/mol*M^-1^)	C_m_ (M)	Δ*G*_D-N_
GdmCl	3.7 ± 0.3	1.6 ± 0.1	5.9 ± 0.7
Urea	2.0 ± 0.2	3.3 ± 0.1	6.6 ± 0.5

Data for Urea and GdmCl were both fit with a two state equation.

Errors are reported as standard deviation.

To obtain a detailed view of the folding free energy landscape, the kinetics of IL-33 were assessed with intrinsic tryptophan fluorescence spectroscopy. The chevron plots are representative of the rates of unfolding and refolding (log *k*_u_ and log *k*_f_) plotted against the respective denaturant concentrations (Fig 2A and 2B, top panel). A single phase is observed in unfolding conditions and through the transition region whereas two phases each with rollover, or a systematic deviation from a linear dependence of folding rate on denaturant concentration, are observed in strongly native conditions. The *m*_D-N_ values of IL-33 (Table 2) obtained from the fits of the chevron plots within the linear regime are comparable with the two-state equilibrium fit *m*_D-N_ values (Table 1), although the parallel phases lead to some mis-match between the Cm values as is seen in other multi-channel folders [25,26]. The chevron plots were fit to the linear regime at ± 2M from the midpoint (C_m_) to highlight the distinct rollover. This systematic deviation from linear dependence on denaturant concentration is indicative of more complicated folding kinetic mechanism.

**Table 2:** Kinetic Data.

	log *k*_f_^H2O^ (s^-1^)	*m*_f_ (M^-1^)	log *k*_u_^H2O^(s^-1^)	*m*_u_ (M^-1^)	*β*_*T*_	C_m_ (M)	*m*_D-N_ (kcal/mol*M^-1^)	log *K* ^H2O^(s^-1^)	Δ*G*_D-N_
GdmCl	0.8±0.1	-1.9± 0.1	-5.2±0.1	1.2±0.1	0.4±0.1	1.9 ±0.1	3.1±0.1	6.0±0.4	8.0± 0.4
Urea	0.7±0.1	-1.0±0.1	-5.5±0.2	0.6±0.1	0.4±0.2	4.1±0.1	1.6±0.1	6.2±0.4	8.2 ± 0.4

Data for Urea and GdmCl were both fit at ± 2M of the midpoint.

Errors are reported as standard deviation.

In order to fully evaluate the source of the rollover in IL-33, a thorough investigation of known and well-established sources that can lead to this kinetic behavior was performed [27–29]. Protein aggregation, proline isomerization, and oligomerization from disulfides were evaluated and showed no significant effects on the refolding rates of IL-33 (S2 Fig). As evidenced by the similar rollover in both GdmCl and Urea, we can conclude that it is not an effect of electrostatics (Fig 2A and 2B, top panels).

**Fig 3:**
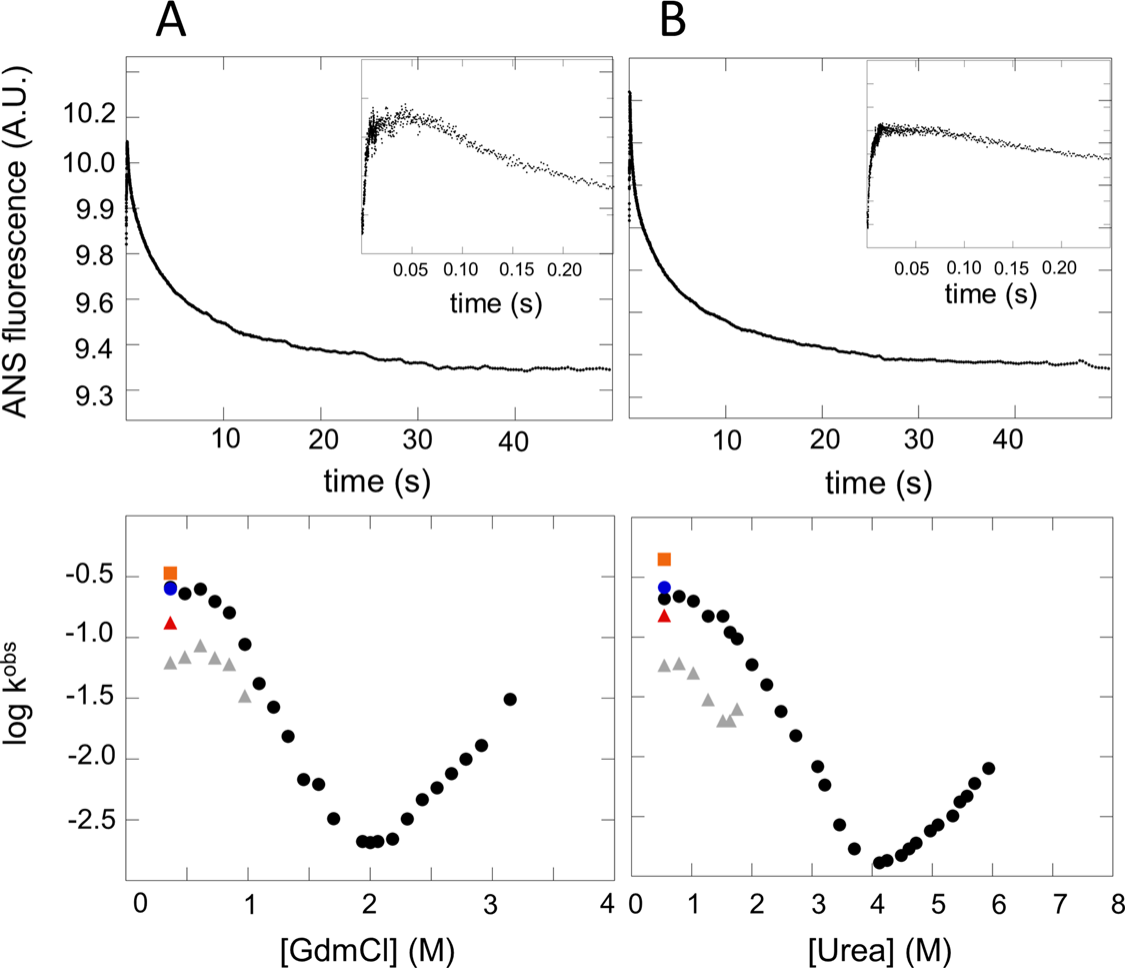
ANS binding kinetics to Interleukin-33 for the refolding reaction. (A, upper panel) A refolding trace of IL-33 in the presence of 1-Anilinonaphtalene-8-Sulfonic Acid (ANS) in 0.36 M GdmCl. (B, upper panel) Refolding of IL-33 in the presence of ANS in 0.36 M urea. Fluorescence emission of ANS was monitored upon refolding of IL-33 and shows an initial binding and release of dye in the first milliseconds of folding (inset), indicative of the formation of a short-lived intermediate state. (A, lower panel) shows the rates of refolding of IL-33 in GdmCl as determined by ANS on the corresponding chevron plot and (B, lower panel) shows the rate of IL-33 refolding in Urea as determined by ANS on the corresponding chevron plot. The ANS binding rate is shown with an orange square while the two phases of ANS release are shown with a blue circle and red triangle, respectively.

The presence of a rollover in the refolding arm of the chevron plots of IL-33 is indicative of an intermediate state, yet there was no direct signature from the intrinsic fluorescence kinetic traces of an intermediate state accumulation. To directly probe for the formation of an intermediate state, IL-33 was refolded in the presence of ANS (1-Anilinonaphtalene-8-Sulfonic Acid), a dye known to bind transient hydrophobic patches in the refolding of proteins [27]. IL-33 was refolded with ANS into strongly native conditions from both GdmCl and Urea (Fig 3). Refolding traces show the formation of an intermediate state populated in the first milliseconds of folding (inset, Fig 3), as evidenced by the increase in fluorescent signal of ANS followed by signal decay as IL-33 folds to the native state and dye is released. Fitting the rate of refolding of IL-33 as a function of ANS release as a double exponential equation showed good agreement for both phases present in the refolding rollover regime (Fig 3, bottom panels). The rate of intermediate formation proved to be fast in the strongly native conditions of the rollover region and slower dependence on denaturant concentration (S3 Fig). Collectively, the experimental data shows that IL-33 folds with three-state kinetics mediated by the presence of an intermediate state. Unfortunately, the intermediate state is difficult to isolate experimentally due to its rapid formation and short existence of this species. Additionally, IL-33 has fast dynamics within the native basin making it non-conducive to analyze a folding intermediate through pulse labeling analysis. So in order to analyze the folding route of IL-33, coarse-grained structure-based simulations were used to provide a more detailed view of the folding free energy landscape.

### Characterizing the Details of the Interleukin-33 Folding Landscape Using Structure Based Models

**Fig 4:**
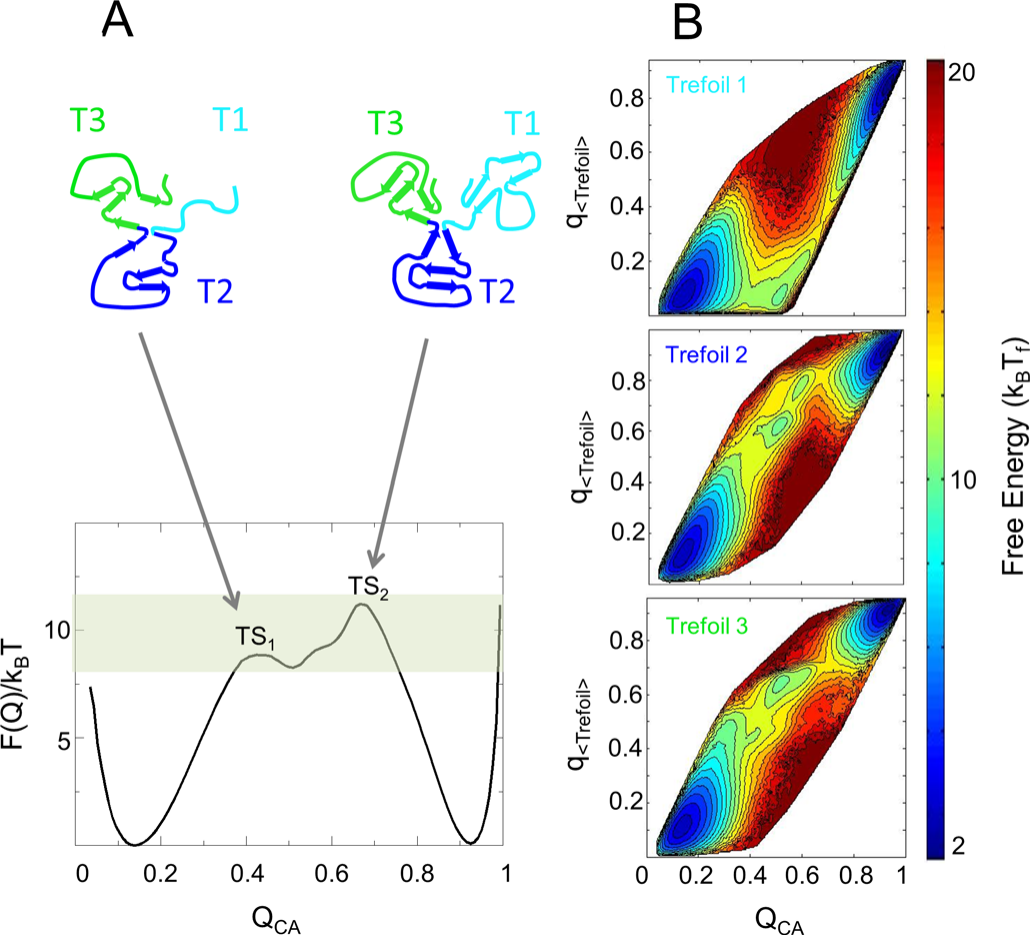
Free energy profile of Interleukin-33 and its individual trefoils determined from structure-based simulations. (A) A plot of the free energy as a function of Q_CA_. The denatured basin is populated at a Q_CA_ of 0.2, the native basin is populated at a Q_CA_ of 0.9 and the transition region has two transition states (TS) at Q_CA_ of 0.4 (TS_1_) and at Q_CA_ of 0.7 (TS_2_). The shaded region highlights the transition state region for IL-33. Representative structures of the species present at each of the two transition states are shown above the profile plot. (B) Two dimensional free-energy landscapes as a function of q_<trefoil>_ and Q_CA_ for Trefoils 1, 2 and 3. The occupancy of states is represented by a color scale where red represents lowest occupancy and blue represents highest occupancy.

Coarse-grained C_alpha_ (CA) structure-based models (SBM) were used to determine the molecular mechanisms behind the kinetics observed from experiments. SBM are well-suited for discerning topological aspects of the folding landscape [28]. The energy landscape of the SBM was exhaustively sampled to obtain thermodynamic information. In Fig 4, the free energy landscape F(Q_CA_)/k_B_T is plotted as a function of the overall folding reaction coordinate Q_CA_, representing the fraction of total native contacts formed on the folding free energy landscape. At the folding midpoint (C_m_) where the probability of populating the denatured and native state is equal (T = T_f_), IL-33 has a broad barrier between 0.3< Q_CA_ <0.8 that contains a low populated intermediate with a depth of < 1 k_B_T. On either side of the intermediate state are two transition states (TS), TS_1_ and TS_2_ at a Q_CA_ of 0.4 and 0.7, respectively. At the folding midpoint, TS_2_ is the rate-limiting transition with a barrier of 11 k_B_T, whereas TS_1_ has a barrier of 9 k_B_T.

**Fig 5:**
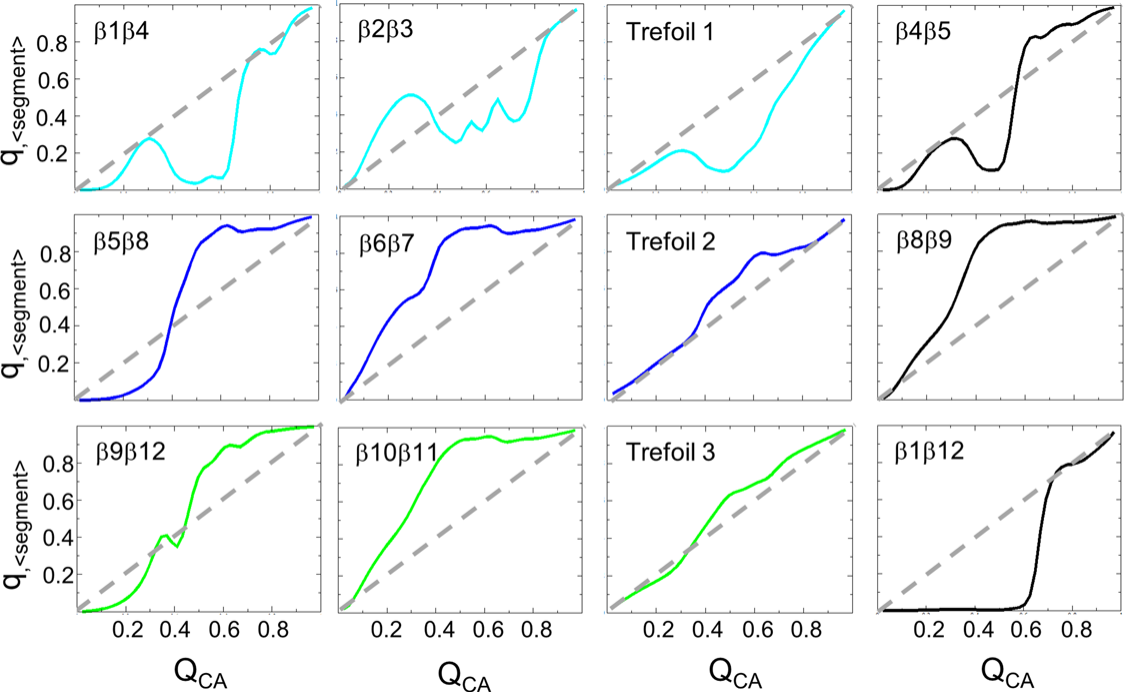
The formation of individual contacts represented for each individual trefoil in Interleukin-33. Plots of q_<segment>_ versus Q_CA_ for interactions between individual strands or within trefoil units are represented. Each plot represents the formation of contacts for the given strands (q_<segment>_) versus the formation of all native contacts within IL-33 (Q_CA_). The plots for contacts located in Trefoil 1 are highlighted in cyan, Trefoil 2 in blue, and Trefoil 3 in green. The contacts formed between trefoils are highlighted in black. The dashed lines compare the overall foldedness of the protein.

As the protein folds, the structural pseudo-symmetry is broken, leading to a specific folding order for the trefoils. The formation of the three trefoils are separated in Fig 4B. The two dimensional free energy landscapes show that successful folding of Trefoil 1 occurs later than Trefoils 2 and 3. Comparing these 2D landscapes with the 1D landscape shows that TS_1_ consists of an intermediate state composed of Trefoils 2 and 3 forming preliminary contacts (q_<trefoil>_ = 0.4) while Trefoil 1 remains largely unfolded. TS_2_ involves the final folding of Trefoil 1 onto the fully formed transition state. Taken together, the data indicates that this symmetry breaking between the trefoils leads to an intermediate state characterized by a majority of contacts in Trefoils 2 and 3 being formed while Trefoil 1 is largely unfolded. Analyzing the structural components in more detail, Fig 5 shows the formation of each β-strand interface (q_<segment>_) relative to the overall foldedness (Q_CA_) of the protein. The contacts in Trefoil 2 and 3 grow monotonically with Q_CA_, with pronounced increases near a Q_CA_ of 0.4, corresponding to TS_1_. In contrast, native contact formation in Trefoil 1 grows until a Q_CA_ of 0.35, but then the contacts are broken before reaching TS_1_. These broken contacts in Trefoil 1 are then reformed at a Q_CA_ of 0.7. Within Trefoil 1, there is complete backtracking of the contacts formed between β-strands 1 and 4 and partial backtracking between β-strands 2 and 3 (Fig 5). The landscape indicates that if Trefoil 1 folds early it tends to backtrack in order to follow the lowest free-energy route to the folded state. Individual strand formation is shown in S4 Fig Interestingly, since unfolding occurs on the same landscape, this means that Trefoil 1, the functional binding element, is the first to unfold coming from the native basin. This architecture could provide malleability to the binding interface, allowing it to be responsive to receptor binding without significantly perturbing the rest of the stable protein scaffold.

## Discussion

The recent discovery of IL-33 and its prevalence in the pathology of multiple disease states [29–31] places it as a central target in drug design. As a member of the β-trefoil family, IL-33 has a unique trefoil topology represented by a pseudo-symmetrical fold. The binding of the Trefoil 1 interface to ST2 is the critical initiating step in the formation of an active signaling heterotrimeric complex, therefore Trefoil 1 represents the functionally critical element of IL-33 [15]. Understanding the folding route of IL-33, specifically the folding of Trefoil 1, can highlight possible underlying sources of disease states by identifying folding events critical for the formation of the functional native state.

In evaluating the folding free energy landscape of IL-33, the chevron plots provide initial evidence for the formation of an intermediate state due to the presence of the rollover in strongly native conditions. However, the rollover only provides indirect evidence of an intermediate state. The intermediate state for IL-33 proved to be difficult to isolate using intrinsic fluorescence due to its short residence time and fast folding from the intermediate state ensemble to the native state. The refolding assay of IL-33 with ANS showed binding and release of dye in the millisecond timescales (Fig 3, inset), providing direct evidence for the first time of an intermediate state in the folding of IL-33. Interestingly, Hisactophilin and hFGF-1, two other β-trefoil proteins, exhibit rollover in their chevron plots in strongly native conditions [32,33] attributed to an intermediate state. The kinetic rates of refolding for IL-33 obtained from analysis using ANS showed agreement between both phases in the rollover region in the chevron plots. We hypothesize that the second slower phase of refolding could be a result of parallel refolding routes that are dependent upon the adjustment of contacts as the geometrically frustrated Trefoil 1 docks onto Trefoils 2 and 3, particularly as Trefoil 1 is constrained to a specific geometric orientation for folding to the native basin. Additionally, the folding of the β-trefoil cytokine Interleukin-1β has also been found to fold through a three-state mechanism [20,21]. In gaining an understanding of how the formation of an intermediate state dictates the steps along the folding route and the interplay of functional elements, we can better understand the functional motions inherent to the activity of native IL-33.

### The Implications of Symmetry on the Folding Landscape

Symmetry in proteins often leads to complicated folding kinetics and a geometrically-frustrated landscape [21,34]. The effects of pseudo-symmetry on the β-trefoil fold [20,35,36] has been studied in terms of both folding and function [28,37,38]. The balance between minimizing the frustration on the folding free energy landscape and preserving the functional regions of a protein is often complex and varied, especially concerning symmetric proteins [39–42]. Satisfying functional constraints can lead to geometric frustration that decreases the folding rate. In the case of IL-33, functional constraints and the interplay of the individual folding elements contribute to both the intermediate species and geometrical frustration in the folding of the functional element of the protein, Trefoil 1.

### Backtracking of the Functional Element, Trefoil 1, Highlights the geometrical Frustration

Backtracking is recognized as a phenomenon in protein folding wherein native contacts are made, broken, and reformed in order to reestablish productive folding along the folding route [43,44]. Here, we observe backtracking in IL-33, in that the symmetry of IL-33 allows all trefoils to locally fold in the denatured state, but only Trefoils 2 and 3 have more than half of their contacts formed in TS_1_. Therefore, if Trefoil 1 folds and forms native contacts early it tends to backtrack in order for IL-33 to follow the lowest free-energy route to the folded native state. We suggest that the backtracking within Trefoil 1 serves to reduce the frustration within Trefoils 2 and 3, leading to successful folding of these Trefoils first, followed by the final folding of Trefoil 1.

**Fig 6:**
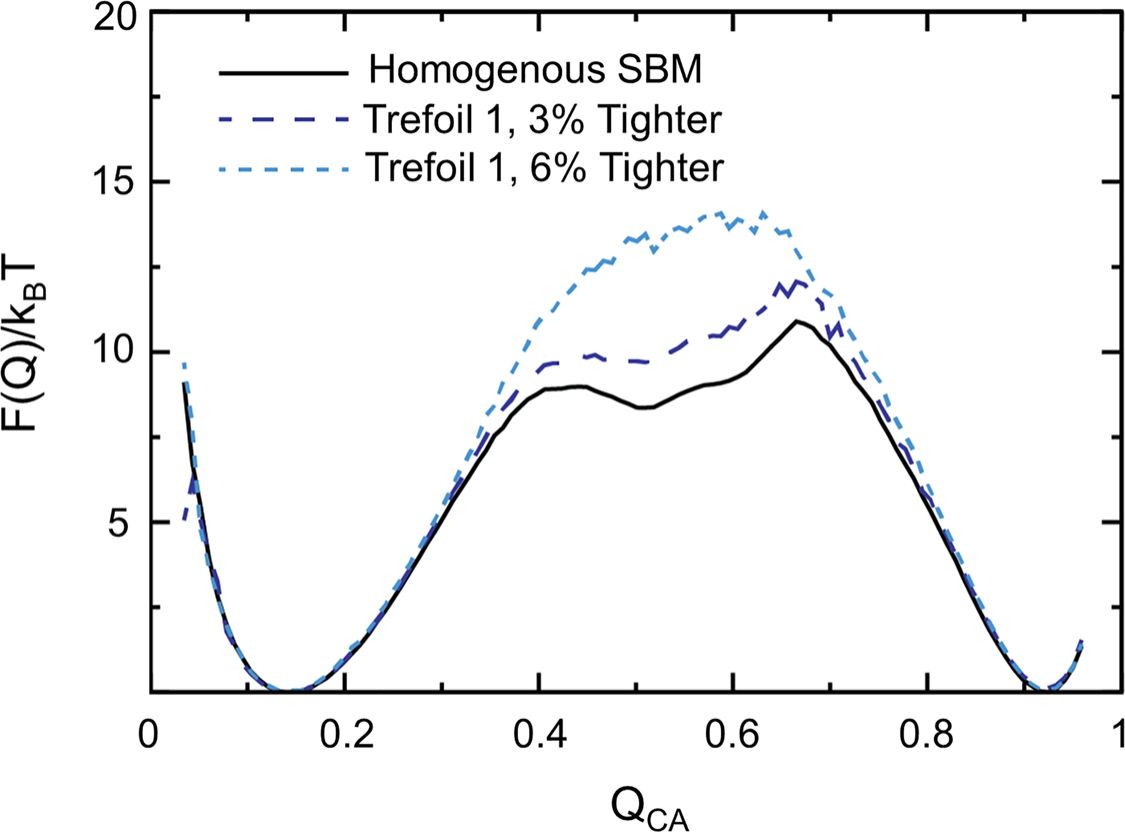
Inducing Cooperative folding through tightening contacts creates an unfavorable two-state folding behavior. The free energy profile is represented as Q_CA_ versus F(Q_CA_)/k_B_T. The wild-type IL-33 is represented in black and IL-33 with tighter contacts introduced in Trefoil 1 (ε^*i*,*j*^ = ε*1.03) is represented in dark blue while (ε^*i*,*j*^ = ε*1.06) is represented in light blue. IL-33 with tightened contacts in Trefoil 1 induces two-state cooperative folding as evidenced by the presence of only one transition state. Additionally, this two-state folding is unfavorable with respect to native three-state folding as evidenced by the larger barrier to the native state, slowing the folding process.

Revisiting the topology of IL-33, the trefoils constitute three individual folding elements that combine to form the final native structure. The intermediate state is composed of partially folded Trefoils 2 and 3, which then act as a scaffold for the folding and docking of Trefoil 1 along with the final folding of Trefoils 2 and 3 during the final rate-limiting step to fold to the native state. To test our hypothesis that Trefoil 1 creates geometric frustration, all contacts were strengthened in Trefoil 1 to evaluate how artificially enhancing the early folding of Trefoil 1 influenced the folding landscape (Fig 6) With a small perturbation (increasing the strength of contacts involving Trefoil 1 by 6%), IL-33 folds through a two-state route, however, the energetic barrier increases dramatically. The increase of the folding barrier demonstrates that the folding of Trefoil 1 cooperatively with Trefoils 2 and 3 is unfavorable and much slower. Breaking the symmetry in IL-33 allows it to fold non-cooperatively and introduces backtracking and an intermediate state on the free energy landscape. Although this type of landscape often leads to slower folding of a protein, in the case of IL-33, the modular folding reduces the folding barrier allowing for both faster folding and local motions important for function.

### Is the Observed Kinetic Rollover Caused by a Moving Rate-Limiting Transition State?

**Fig 7:**
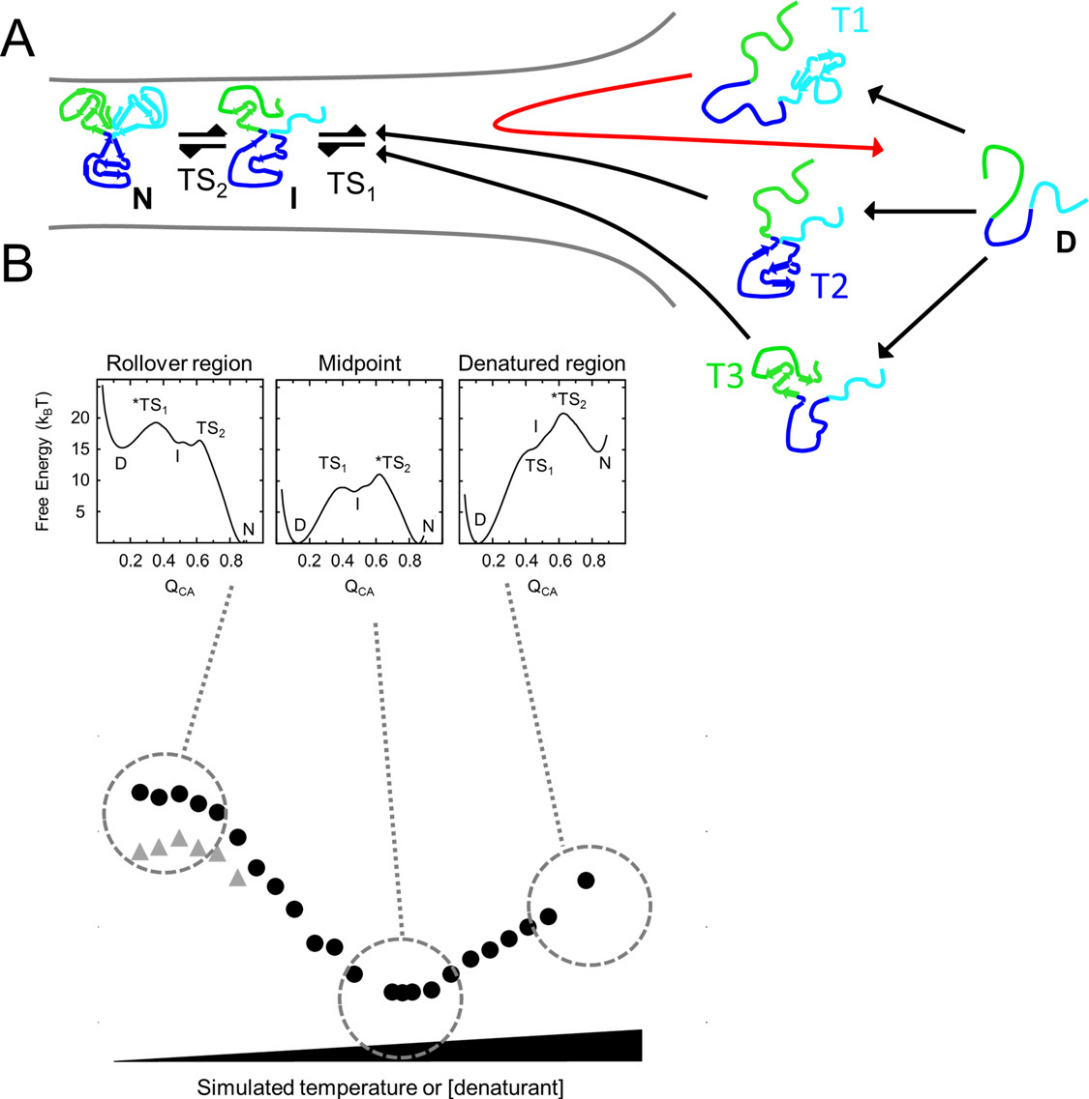
Position of rate-limiting transition state changes with protein stability. (A) Representative schematic of the folding landscape of IL-33. From left to right represents the transition from the native to the denatured state of the protein as seen in the presence of low to high simulated temperature or experimental from low to high denaturant concentration. The intermediate state populated on the folding route is composed of Trefoils 2 and 3, where most contacts are formed, while Trefoil 1 remains unfolded. Folding of Trefoils 2 or 3 can lead to productive folding to the intermediate state along parallel routes, whereas folding of Trefoil 1 first leads to backtracking and non-productive folding (red arrow). (B) Geometric frustration on the folding landscape of IL-33 creates two bottlenecks to fold (TS_1_ and TS_2_) where an intermediate ensemble can be populated. Under folding conditions, TS_1_ is the rate-limiting step, while closer to the midpoint and beyond, TS_2_ is rate-limiting. The position of TS1 and TS2 along the reaction coordinate is affected differentially by denaturant, as the denaturant effect is strongly related to the change in solvent accessible surface area (SASA). The second transition state ensemble has a larger SASA than the first transition sate ensemble, meaning that denaturant should have a larger effect (larger slope) in the regime where TS_2_ is rate-limiting.

As seen in the simulation-derived free-energy profile of IL-33 (Fig 4A), there are two observable transition states, TS_1_ and TS_2_, along the folding route. These transition states are positioned on either side of the intermediate species. Movement of the rate-limiting TS is a canonical case of chevron rollover, as seen in U1A [45]. As temperature increases in the simulation (a proxy for denaturant in the experiment), a Hammond [46] effect causes the rate-limiting TS to move from having little structure, a Q_CA_ of 0.4, to significant structure, a Q_CA_ of 0.7 (Fig 7). A transition state with low Q_CA_ means it has little change in SASA relative to the denatured ensemble (**Δ**SASA). Since denaturant lowers the free energy in a configuration roughly proportional to **Δ**SASA, denaturant will have less effect on an early transition state, leading to a smaller slope in the chevron plots (Fig 2A and 2B, top panel). The intermediate state, where Trefoils 2 and 3 are partially folded and Trefoil 1 is largely unfolded, has a shallow denaturant dependence due to the limited buried surface area present. In TS_2_ where larger portions of the protein become folded, **Δ**SASA increases and the denaturant dependence of the refolding reaction increases, leading to the sharpening of the slope of the refolding arm. Thus, the rollover in the chevron plot, at low denaturant, is consistent with the energy landscape calculated from simulations (Fig 7). Within the rollover region, the transition state barriers TS_1_ (from D to I) and TS_2_ (from I to N) are well separated, suggesting that there is broadness to the reaction coordinate where the intermediate state can reside (Fig 7B). This means that the intermediate state can either cross TS_2_ at a Q_CA_ of 0.7 to fold to its native state or that it may cross TS_1_ to the denatured basin. Both transition state barriers require energy to cross and thus allow for the existence of an intermediate in strongly native conditions, at lower GdmCl concentrations. The formation of the intermediate state is slowed as the protein is refolded in higher GdmCl concentrations, closer to the midpoint. We hypothesize that this behavior could be a result of the switch of the rate-limiting step from TS_1_ to TS_2_, allowing for a slower refolding rate of the intermediate state in the transition region as the folding reaction progresses toward the midpoint (S4 Fig).

## Conclusions

Through a combination of experimental and theory-based work we present that the modular folding and the functionality of IL-33 are intricately related. The intermediate state of IL-33 is characterized by the folding of a metastable intermediate state composed of partially folded Trefoils 2 and 3 and a largely unfolded Trefoil 1. The formation of the intermediate state allows for the subsequent folding of the geometrically frustrated Trefoil 1 as the final step to reach the native fold. Even though all trefoils share similar (but not identical) contacts, similar structural elements, and similar geometry, Trefoil 1 folds differently and has differential effects on the functionality of the protein. Thus Trefoil 1 can respond, function, and be malleable without fully unfolding the protein. Targeting the intermediate formation of Trefoils 2 and 3 and preventing the final folding of Trefoil 1 could be a good strategy to prevent binding to the receptor and subsequent signaling. Further, due to the increased stability of Trefoils 2 and 3 with respect to Trefoil 1, the more stable scaffold of Trefoils 2 and 3 would be an excellent drug target. This further implies that Trefoil 1 can respond to changes caused by engaging the binding interface of its cognate receptor without dramatically altering the protein scaffold.

## Materials and Methods

### Sample Preparation

IL-33 was expressed in a Pet24 vector (Novagen) and grown in BL21 cells (Novagen). Initial purification was achieved using osmotic shock cell lysis and final purity was attained through anion exchange chromatography. All experiments were performed in 10 mM MES, 90 mM NaCl, 1 mM EDTA at pH 6.50.

### Equilibrium Measurements

Equilibrium titrations were collected on a FluoroMax-4 at 25°C. Excitation was at 280 nm and emission was monitored between 350 and 450 nm. The final protein concentration was 5 μM.

### Kinetic Measurements

Refolding kinetics were performed on a PiStar-180 stopped flow fluorometer at 25°C. Excitation was at 280 nm and data was collected using a 320 nm cut off filter. Final protein concentration was 1 μM. Unfolding kinetics were monitored on a FluoroMax-4 using manual mixing with anti-photo bleaching due to hyper fluorescence upon unfolding. Folding was fit to a three-state fit at low denaturant concentrations (below 1.3M for GdmCl and below 1.8M for Urea) and all other data was fit to a two-state fit. We evaluated the chevron plots with a linear fit at ±2M from the midpoint to minimize denaturant dependence. ANS was excited at 365 nm and data was collected with a 420 nm cut off filter. The denatured protein was incubated with ANS at a final concentration of 100 μM and refolded into 0.36 M denaturant and 1 M denaturant, respectively.

### Structure Based Models (SBM)

In this study we used a structure-based Cα model to investigate the folding of IL-33 [47,48]. Each residue is assigned as a single bead. These beads are assigned attractive interactions based upon their relative proximity to one another in the native state. Native contacts are determined using a shadow map [49,50], which relates relative distances and possible steric interferences to extrapolate a reasonable cutoff for native contacts. The basic Hamiltonian is:

$$\begin{array}{l} V(r_{ij})=\sum_{\text{bonds}\{ij\}}k_{b}{\left(r_{ij}-r_{ij}^{N}\right)}^{2}+\sum_{\text{angles}\{ijk\}}k_{a}{\left(\theta_{ijk}-\theta_{ijk}^{N}\right)}^{2}+\\ \sum_{\text{dihedrals}\{ijkl\}}k_{d}^{1}\left[1-\text{cos}{\left(\varphi_{ijkl}-\varphi_{ijkl}^{N}\right)}^{2}\right]+k_{d}^{3}\left[1-\text{cos}{\left(3(\varphi_{ijkl}-\varphi_{ijkl}^{N})\right)}^{2}\right]+\\ \sum_{\text{native contacts}}R(r_{ij})+G_{ij}(r_{ij})+R(r_{ij})G_{ij}(r_{ij})+\sum_{\text{nonnative contacts}}R(r_{ij})\\ R(r_{ij})=\varepsilon {\left(\frac{\sigma }{r_{ij}}\right)}^{12}\\ G_{ij}(r_{ij})=-\varepsilon \;\text{exp}\left(-{\left(r_{ij}-r_{ij}^{N}\right)}^{2}/2w^{2}\right) \end{array}$$

Native interactions have a repulsive term plus an attractive Gaussian term. The R(r_*ij*_)G_*ij*_(r_*ij*_) term is a correction that anchors the minimum of each contact -e (where the last two corresponds respectively to attractive and repulsive non-bonded interactions [51]. $r_{ij}^{N}$ denotes the native distance between atoms *i* and *j* along the sequence. The local topology of the chain is described by the native angles $\theta_{ijk}^{N}$ between the bonds connecting residue pairs *ij* and *jk*, and by the native dihedrals $\phi_{ijkl}^{N}$ or torsional angles between the planes defined by atoms *ijk* and *jkl*. The strengths of the interactions are given in reduced energy units by the constants k_b_ = 2 x 10^4^ e/nm^2^, k_a_ = 40 e/rad^2^, k^1^_d_ = e and k^2^_d_ = 0.5e, where e is the reduced energy unit. The details of the model are characterised elsewhere [28,51].

The webserver SMOG (http://smog-server.org/) was used to create the input files for our simulations. All simulations were performed using GROMACS 4.5.3 software package [52]. Integration steps of t = 0.005 were used in all simulations and all results are presented with reduced units. For sufficient sampling of the transition state, IL-33 was simulated using umbrella sampling along the coordinate Q_CA_ [53]. In order to analyze the correct folding mechanism, (to create F(Q_CA_) for the free energy profiles) we used the Weighted Histogram Analysis Method (WHAM) [54,55]. The results are reported as units of ε, representative of the energy gain in forming a native contact. In the case of the tightened contacts in Trefoil 1, the contacts between *i* and *j* were tightened by ε^*i*,*j*^ = ε*1.03 and 1.06 and the rest of the contacts were homogenously reduced to keep the total stabilizing energy constant.

## Supporting Information

S1 Fig Fits of refolding kinetic traces of Interleukin-33 and the associated residual plots. (A and B) Shows the refolding trace of IL-33 into 0.36 M GdmCl in black with the double exponential fit in red (A) and the refolding trace of IL-33 into 0.54 M Urea in black with the double exponential fit in red (B). (A and B, inset) Shows the residuals of a single exponential fit (black) and a double exponential fit (red) for the refolding trace of IL-33 into 0.36 M GdmCl (A) and 0.54 M urea (B), points representative of the rollover region in the chevron plots.
(TIFF)

S2 Fig Assessing the effect of concentration, prolyl isomerase, and reducing agents on Interleukin-33 kinetics. (A) Represents the effect of protein concentration from a range of 0.25 μM to 10 μM are plotted onto the chevron plot in the rollover region. There is no significant effect of protein concentration on the rate of refolding. (B) Represents the concentration dependence in the same range as seen in panel A but in the linear portion of the chevron plot. There is no significant effect on the rate of refolding. (C) Represents the effect of reducing agent on the refolding rate of IL-33 in the rollover region. There is no significant effect of denaturant on the rates of refolding. (D) Represents the effect of Cyclophilin D on the rate of refolding in both the rollover and linear portion of the chevron plot. There is no effect of Cyclophilin D from the range of 0 to 2 μM on the rate of folding.
(TIFF)

S3 Fig Denaturant dependent formation of the intermediate state in Interleukin-33. The fits of the intermediate formation in the presence of ANS are plotted as a function of time (s) and fluorescence A.U. of the dye. Black represents IL-33 refolded into 0.36 M GdmCl (the rollover region) while red represents IL-33 folded into 1 M GdmCl (the linear regime). Both were fit to a double exponential fit. The formation of the intermediate is slowed significantly from a dominant rate of log *k*_f_ = -0.48 when refolded into 0.36M GdmCl to a rate of log *k*_f_ = -0.95 when refolded into 1M GdmCl.
(TIFF)

S4 Fig The formation of contacts represented for each individual β-strand in Interleukin-33. Plots of q_<β-strand>_ versus Q_CA_ for interactions between individual β-strands are represented. Each plot represents the formation of contacts for the given strands (q_<β-strand>_) versus the formation of all native contacts within IL-33 (Q_CA_). The plots for contacts located in Trefoil 1 are highlighted in cyan, Trefoil 2 in blue, and Trefoil 3 in green. The dashed lines represent constant growth of elements along the free energy landscape. The β-strands composing Trefoil 3 (β-strands 9 through 11) are the first to form, with pronounced growth at a Q_CA_ of 0.3. The β-strands composing Trefoil 2 (β-strands 5 through 8) form almost concurrently with the β-strands composing Trefoil 3. The β-strands composing Trefoil 1 (β-strands 1 through 4) form and are then subsequently broken before a Q_CA_ of 0.4. The β-strands then pause their growth until after a Q_CA_ of 0.6, indicating that the β-strands in Trefoil 1 will not fold until the β-strands from Trefoils 2 and 3 are nearly folded to completion creating a scaffold for Trefoil 1. The final step in folding is the formation of contacts in β-strand 12.
(TIFF)
