# Supplementary figures and images for: Geometrical Frustration in Interleukin-33 Decouples the Dynamics of the Functional Element from the Folding Transition State Ensemble
Source: PLoS One. 2015 Dec 2;10(12):e0144067. doi: 10.1371/journal.pone.0144067 (PMC4667907; doi:10.1371/journal.pone.0144067)

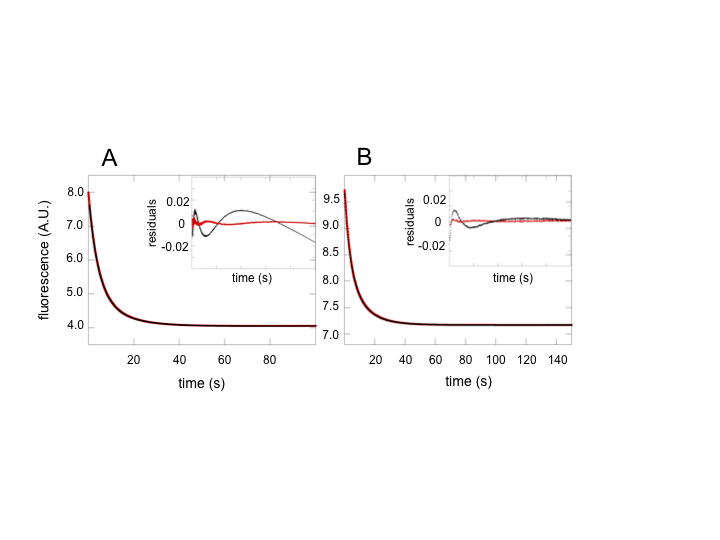

Supplement: S1 Fig — (A and B) Shows the refolding trace of IL-33 into 0.36 M GdmCl in black with the double exponential fit in red (A) and the refolding trace of IL-33 into 0.54 M Urea in black with the double exponential fit in red (B). (A and B, inset) Shows the residuals of a single exponential fit (black) and a double exponential fit (red) for the refolding trace of IL-33 into 0.36 M GdmCl (A) and 0.54 M urea (B), points representative of the rollover region in the chevron plots. (TIFF) [file pone.0144067.s001.tiff]

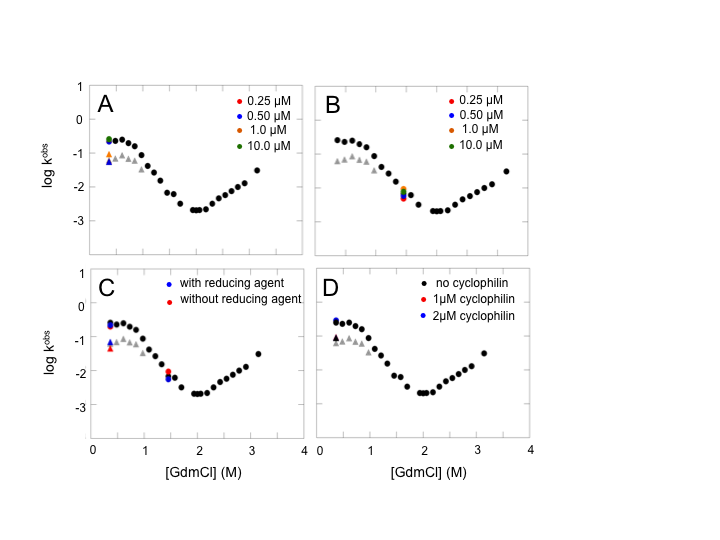

Supplement: S2 Fig — (A) Represents the effect of protein concentration from a range of 0.25 μM to 10 μM are plotted onto the chevron plot in the rollover region. There is no significant effect of protein concentration on the rate of refolding. (B) Represents the concentration dependence in the same range as seen in panel A but in the linear portion of the chevron plot. There is no significant effect on the rate of refolding. (C) Represents the effect of reducing agent on the refolding rate of IL-33 in the rollover region. There is no significant effect of denaturant on the rates of refolding. (D) Represents the effect of Cyclophilin D on the rate of refolding in both the rollover and linear portion of the chevron plot. There is no effect of Cyclophilin D from the range of 0 to 2 μM on the rate of folding. (TIFF) [file pone.0144067.s002.tiff]

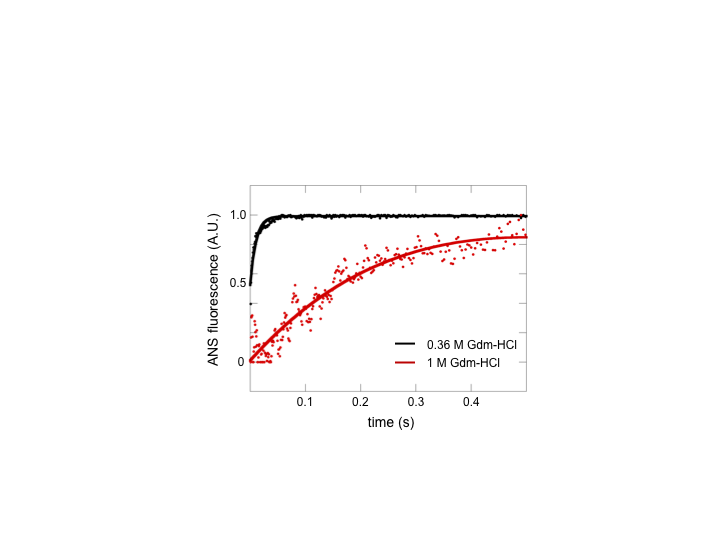

Supplement: S3 Fig — The fits of the intermediate formation in the presence of ANS are plotted as a function of time (s) and fluorescence A.U. of the dye. Black represents IL-33 refolded into 0.36 M GdmCl (the rollover region) while red represents IL-33 folded into 1 M GdmCl (the linear regime). Both were fit to a double exponential fit. The formation of the intermediate is slowed significantly from a dominant rate of log k f = -0.48 when refolded into 0.36M GdmCl to a rate of log k f = -0.95 when refolded into 1M GdmCl. (TIFF) [file pone.0144067.s003.tiff]

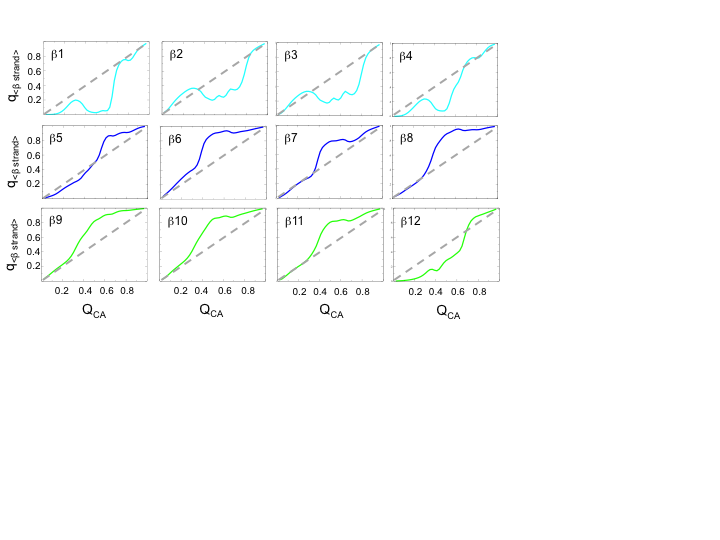

Supplement: S4 Fig — Plots of q<β-strand> versus QCA for interactions between individual β-strands are represented. Each plot represents the formation of contacts for the given strands (q<β-strand>) versus the formation of all native contacts within IL-33 (QCA). The plots for contacts located in Trefoil 1 are highlighted in cyan, Trefoil 2 in blue, and Trefoil 3 in green. The dashed lines represent constant growth of elements along the free energy landscape. The β-strands composing Trefoil 3 (β-strands 9 through 11) are the first to form, with pronounced growth at a QCA of 0.3. The β-strands composing Trefoil 2 (β-strands 5 through 8) form almost concurrently with the β-strands composing Trefoil 3. The β-strands composing Trefoil 1 (β-strands 1 through 4) form and are then subsequently broken before a QCA of 0.4. The β-strands then pause their growth until after a QCA of 0.6, indicating that the β-strands in Trefoil 1 will not fold until the β-strands from Trefoils 2 and 3 are nearly folded to completion creating a scaffold for Trefoil 1. The final step in folding is the formation of contacts in β-strand 12. (TIFF) [file pone.0144067.s004.tiff]
